# Comprehensive Analysis of Replication Origins in *Saccharomyces cerevisiae* Genomes

**DOI:** 10.3389/fmicb.2019.02122

**Published:** 2019-09-13

**Authors:** Dan Wang, Feng Gao

**Affiliations:** ^1^Department of Physics, School of Science, Tianjin University, Tianjin, China; ^2^Key Laboratory of Systems Bioengineering, Ministry of Education, Tianjin University, Tianjin, China; ^3^SynBio Research Platform, Collaborative Innovation Center of Chemical Science and Engineering, Tianjin, China

**Keywords:** replication origin, DNA replication, *Saccharomyces cerevisiae*, genome-wide analysis, autonomously replicating sequence

## Abstract

DNA replication initiates from multiple replication origins (ORIs) in eukaryotes. Discovery and characterization of replication origins are essential for a better understanding of the molecular mechanism of DNA replication. In this study, the features of autonomously replicating sequences (ARSs) in *Saccharomyces cerevisiae* have been comprehensively analyzed as follows. Firstly, we carried out the analysis of the ARSs available in *S. cerevisiae* S288C. By evaluating the sequence similarity of experimentally established ARSs, we found that 94.32% of ARSs are unique across the whole genome of *S. cerevisiae* S288C and those with high sequence similarity are prone to locate in subtelomeres. Subsequently, we built a non-redundant dataset with a total of 520 ARSs, which are based on ARSs annotation of *S. cerevisiae* S288C from SGD and then supplemented with those from OriDB and DeOri databases. We conducted a large-scale comparison of ORIs among the diverse budding yeast strains from a population genomics perspective. We found that 82.7% of ARSs are not only conserved in genomic sequence but also relatively conserved in chromosomal position. The non-conserved ARSs tend to distribute in the subtelomeric regions. We also conducted a pan-genome analysis of ARSs among the *S. cerevisiae* strains, and a total of 183 core ARSs existing in all yeast strains were determined. We extracted the genes adjacent to replication origins among the 104 yeast strains to examine whether there are differences in their gene functions. The result showed that the genes involved in the initiation of DNA replication, such as *orc3*, *mcm2*, *mcm4*, *mcm6*, and *cdc45*, are conservatively located adjacent to the replication origins. Furthermore, we found the genes adjacent to conserved ARSs are significantly enriched in DNA binding, enzyme activity, transportation, and energy, whereas for the genes adjacent to non-conserved ARSs are significantly enriched in response to environmental stress, metabolites biosynthetic process and biosynthesis of antibiotics. In general, we characterized the replication origins from the genome-wide and population genomics perspectives, which would provide new insights into the replication mechanism of *S. cerevisiae* and facilitate the design of algorithms to identify genome-wide replication origins in yeast.

## Introduction

DNA replication is a highly orchestrated process, which is tightly controlled to duplicate the genetic materials into both daughter cells (Bell and Labib, 2016). The specific sites where DNA replication initiates and double-stranded DNA starts unwinding are termed replication origins (ORI) (Jacob et al., 1963; Gilbert, 2001). The identification of ORIs has long been a critical issue, which is helpful to elucidate the molecular mechanism of DNA replication.

The base composition asymmetry widely exists in bacterial genomes (Lobry, 1996; Rocha et al., 1999; Zhang and Gao, 2017; Quan and Gao, 2019). Based on this phenomenon, some strategies to predict replication origin of chromosomes (*oriC*s) have been developed, for instance, GC skew (Lobry, 1996), cumulative GC skew (Grigoriev, 1998), skewed oligomers (Salzberg et al., 1998) and Z-curve (Zhang and Zhang, 2014). Considering the distributions of DnaA boxes and the conserved *oriC*s-adjacent genes in different phyla (Mackiewicz et al., 2004; Luo et al., 2018), the web server Ori-Finder (Gao and Zhang, 2008; Luo et al., 2014) has been developed based on the Z-curve method to predict *oriC*s in bacteria.

For eukaryotes, due to the long linear chromosomes, initiation of DNA replication occurs at multiple discrete sites and activates following the specific timing of DNA replication during the S phase (Taylor, 1960; Hand, 1978; Friedman et al., 1997). The characteristics of eukaryotic replication origins are best understood in the budding yeast *Saccharomyces cerevisiae*. Sequences conferring the ability of the autonomous replication on circular plasmid molecule are termed autonomously replicating sequences (ARSs) (Stinchcomb et al., 1981) that are regarded as ORIs in yeast chromosomes (Brewer and Fangman, 1987). Taking ARS1 as an example, it consists of the A element (ARS consensus sequence, ACS) (Marahrens and Stillman, 1992) where the ATP-dependent origin recognition complex (ORC) specifically recognizes and binds (Bell and Stillman, 1992; Li et al., 2018), the B1 element partially involved in ORC-DNA interaction (Duderstadt and Berger, 2008; Li et al., 2018), and the B2 element associated with mini-chromosome maintenance (MCM) proteins (Wilmes and Bell, 2002). ARS1 also contains the binding site for site-specific DNA-binding protein ABF1 (ARS binding factor I) (Diffley and Stillman, 1988), whereas ABF1 is not a universal ARS-binding factor. The experimental methods for identifying ARSs in yeast such as the two-dimensional (2D) gel analysis (Brewer and Fangman, 1987; Newlon et al., 1993), microarray-based approaches (Lee et al., 2007), chromatin immunoprecipitation (ChIP) including microarray (ChIP-chip) (Wyrick et al., 2001) and sequencing (ChIP-seq) (Eaton et al., 2010) as well as deep sequencing approaches (Müller et al., 2013) have provided plentiful accurate and reliable results. However, they are costly and time-consuming.

With the accumulation of experimental data and sequencing genomes, the available databases related to replication origins in yeast such as SGD (Cherry et al., 2012), OriDB (Nieduszynski et al., 2006a), DeOri (Gao et al., 2012) and DNA replication (Cotterill and Kearsey, 2008) have been established and updated, which brings new opportunities to study ORIs in yeast genome via more efficient and faster bioinformatic methods. For example, Breier et al. (2004) developed an Oriscan algorithm to predict ORIs in the *S. cerevisiae* genome utilizing both the ACS motif and its flanking AT-rich region. Consequently, 84% of the top 100 Oriscan predictions matched known ARSs or replication protein binding sites, whereas with the accumulation of predictions, only 56% of the top 350 Oriscan predictions were matched. The result indicated that the algorithm using the similarity to 26 featured origins may limit the discovery of new potential ARSs. The machine learning-based techniques for predicting ORIs in yeast genome have been developed in recent years. Both iRO-3wPseKNC (Liu et al., 2018) and PseKNC2.0 (Dao et al., 2019) web-servers generated the sample formulation based on the mode of PseKNC (pseudo K-tuple nucleotide composition) for describing nucleotide sequences, and utilized the machine learning methods of random forest (RF) and support vector machine (SVM), respectively. Both the web-servers are user-friendly and efficiently performing, whereas direct extraction of the overall ORI sequence information without highlighting the characteristic conservative motifs will undoubtedly dilute the specific features of ORIs, resulting in lowering the prediction accuracy and increasing the false positives. Nieduszynski et al. (2006b) combined the results of ACS motif searches, phylogenetic conservation and microarray data, which enabled the prediction of essential ORIs throughout the *S. cerevisiae* genome. The result of phylogenetic conservation of replication origin sequences among closely related *Saccharomyces* species evidently improves the determination of the genome-wide location of replication origins, which suggested that multi-aspect analysis of replication origin sequences will facilitate the performance of prediction models. Although ORIs are essential for the maintenance of *S. cerevisiae* genome, the yeast chromosome harboring multiple origin deletions has been reported to replicate relatively normally (Dershowitz et al., 2007; Bogenschutz et al., 2014), that is to say, for an individual ORI, it is optional or redundant, which reflects the unexpected flexibility of DNA replication and also implies that there are still a number of potential replication origins to be discovered. Research on replication origins of only one or several strains may provide limited information. In recent years, the accumulation of published *S. cerevisiae* whole genome sequences (Strope et al., 2015; Zhu et al., 2016; Peter et al., 2018) is unprecedented, hence we can not only comprehensively analyze the ORI features at a genome-wide level, but also have the chance to compare the similarities and differences of replication origin sequences among diverse budding yeast strains from a population genomics respective. However, there are no such reports on the analysis of replication origins based on large-scale genomic data so far.

In this study, we firstly summarized and analyzed the characteristics of replication origin sequences in the reference genome of *S. cerevisiae* S288C, including classification of ARSs and the specific features in different types. Then we retrieved 104 genome sequences of budding yeasts with high genome integrity, and built a non-redundant dataset based on the published ARSs of *S. cerevisiae* S288C and supplemented with the confirmed ARSs from OriDB and DeOri databases, which makes it possible for us to conduct a large-scale comparison of replication origin sequences among various yeast strains from a population genomics perspective. By a pan-genome analysis of ARSs among the *S. cerevisiae* strains, we determined the core ARSs existing in all yeast strains. We also analyzed the distribution bias of various ARSs with different conservation along the chromosomes. To explore whether the ARS-adjacent genes are conserved or not, we extracted genes adjacent to replication origins among the 104 yeast strains. Subsequently, we compared the enriched function of genes adjacent to various ARSs with different conservation and attempted to explain the relationships between replication origins and their adjacent genes.

## Materials and Methods

### Strains and Datasets

We retrieved the reference genome sequence of *S. cerevisiae* S288C (version: R64-2-1) as well as 103 well-annotated budding yeast genome sequences with high genome integrity (>95%) from the NCBI FTP site^1^. The annotation of *S. cerevisiae* S288C was downloaded from the SGD FTP site^2^. From literature sources, the information of ecological origins and geographical origins of above *S. cerevisiae* strains were obtained (Strope et al., 2015; Peter et al., 2018) and the datasets of temporal replication of yeast chromosomes were collected (Raghuraman et al., 2001). We also acquired the available ACS sequences from YeastMine database^3^ populated by SGD.

### Evaluating the Sequence Similarity of ARSs

Sequences similarity analysis of ARSs in *S. cerevisiae* S288C reference genome was conducted by local BLAST 2.7.1+ (Camacho et al., 2009) (cutoff: *E*-value ≥ 5e-10, identity ≥90%, coverage ≥90%). Subsequently, the ARS sequences that have multiple alignment results were visualized by ClicO FS (Cheong et al., 2015).

### Scan of Homologous ARSs in 104 *S. cerevisiae* Strains

In this study, a non-redundant dataset consisting of 520 ARSs was constructed based on the available ARSs of *S. cerevisiae* S288C from SGD database (Cherry et al., 2012) and supplemented with the confirmed ARSs from OriDB database (Nieduszynski et al., 2006a)^4^ and ARSs of *S. cerevisiae* from DeOri 6.0 database (Gao et al., 2012)^5^. For each ARS in this dataset, we performed a BLAST against 104 *S. cerevisiae* genomes (cutoff: *E*-value ≥ 5e-10, identity ≥90%, coverage ≥90%). Then, a custom Python script was used to extract the aligned information, which was converted into a GFF3 format file. The conservative profile of ARSs was statistically analyzed according to the frequency of homologous ARSs among 104 *S. cerevisiae* strains. Finally, we divided the annotation file of sorted homologous ARSs into 104 separate annotation files based on strain names.

### Extraction of the Protein-Coding Genes Adjacent to Replication Origins

Firstly, we merged the ARS annotation files with the CDS annotation files of the corresponding 104 *S. cerevisiae* strains. Then, a custom Python script was used to extract the adjacent genes on both sides of ARSs, and the interval or intersected distance between ARSs and their adjacent genes were calculated. Subsequently, the sequences of these genes adjacent to ARSs among 104 *S. cerevisiae* strains were collected to BLAST against the dataset obtained from the latest version (February 2019) of UniProtKB^6^ by the “blastp” program with the *e*-value cutoff of 1e-5.

### Functional Enrichment Analysis

Lists of genes adjacent to conserved ARSs and non-conserved ARSs were prepared through the above steps. Then the gene lists were submitted to DAVID website (Huang et al., 2008) to perform the enrichment analysis of the Gene Ontology (GO) terms and Kyoto Encyclopedia of Genes and Genomes (KEGG) pathways by Fisher’s exact test. False discovery rate (fdr) was used to filter out the false-positive results with the cutoff of 0.05 for statistical significance.

## Results and Discussion

### Characteristic Analysis of ARSs in *S. cerevisiae* Genomes

#### Overview of ARSs in *S. cerevisiae* S288C Reference Genome

There are a total of 352 ARSs in *S. cerevisiae* S288C reference genome available in the SGD database (Supplementary Table 1). The length of these experimentally verified ARSs ranged from 51 to 1324 bp and mainly (63.63%) concentrated in 70–250 bp (Supplementary Figure 1A), and the median length of ARSs in each chromosome is mainly around 240 bp (Supplementary Figure 1B). By linear regression analysis, the fitting line suggested that the count of ARSs was positively correlated to the length of chromosomes with the correlation coefficient of 0.7758 (Supplementary Figure 1C).

The *S. cerevisiae* S288C genome sequence has an average GC content of 38.38%, while that of ORI sequences is only 29.65%. With the accumulation of experimental data, there are 196 published ACS sequences in the YeastMine database populated by SGD. About 87.43% of ARSs that contain the ACS element are within the length of 300 bp. Here, we took the ACS element as the center to explore the base distribution of ARSs (Supplementary Figure 2) by WebLogo plot (Crooks et al., 2004). The ACS is visible as the high central peak with a high proportion of T residues. With the increasing number of ACS subjects, the degenerate ACS is slightly changed from 5′-WTTTATRTTTW-3′ (Broach et al., 1983) to 5′-WTTTAYRTTTW-3′ (Marahrens and Stillman, 1992). In this study, we collected those published 196 ACS sequences from YeastMine and generated the matrix profile of ACS motif (Supplementary File 1) using “Bio.motifs” package included in Biopython for subsequent prediction of ACS in the candidate sequence. A broad region located directly 3′ to the ACS termed B elements (Lucas and Raghuraman, 2003), which showed low sequence similarity among various ARSs. And only minimal conservations located 3′ to the ACS were detected, for example the conserved 5′-TT-3′ of B1 element and a relatively high frequency of adenine residues start from around 30 to 110 bp, which are consistent with the previous research (Broach et al., 1983; Huang and Kowalski, 1996; Lucas and Raghuraman, 2003; Breier et al., 2004).

**FIGURE 1:**
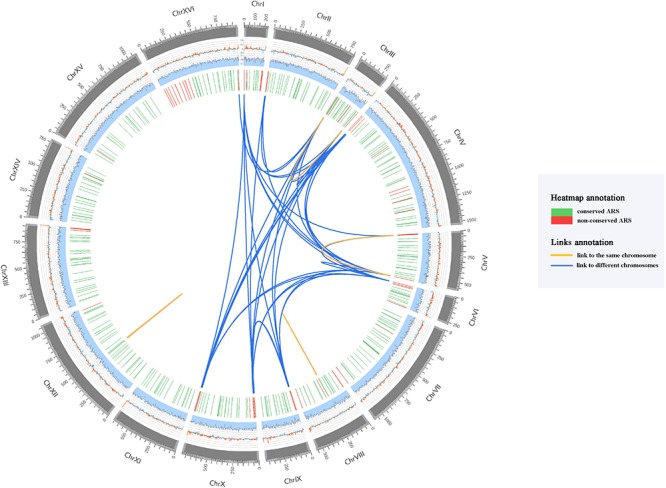
Circos plot showing the ARSs distribution of *S. cerevisiae* S288C reference genome. Every circle is described in the outermost-innermost direction. (1) The outermost circle represents the *S. cerevisiae* S288C chromosomes in kb, and the subtelomeric regions are colored in lighter gray; (2) GC-skew (window=3 kb, step=3 kb); (3) AT content (window=3 kb, step=3 kb); (4) Conservation heatmap of 352 ARSs. The location of each bar in the heatmap denotes the position of the ARS in each chromosome of *S. cerevisiae* S288C, and the number of homologous ARSs in 104 yeast strains is represented by the color of green (conserved ARS) and red (non-conserved ARS); (5) Links showed the result of similar ARS sequences. The orange links represent the similar ARS sequences that are mapped to the identical chromosome, and the blue links represent the similar ARS sequences that are located on different chromosomes.

In order to investigate the uniqueness of the ARS sequences in *S. cerevisiae* S288C yeast genome, we conducted a sequence similarity analysis of all these 352 annotated ARSs. The majority of ARS sequences (94.32%) are unique, while only 20 ARSs have multiple alignment results (Supplementary File 2) distributed in intra-chromosomes and inter-chromosomes (Figure 1). Interestingly, all the similar ARS pairs distributed in inter-chromosomes are biased to locate in subtelomeric regions generally within 20 kb of both ends of yeast chromosome (Brown et al., 2010). Yue et al. (2017) found that subtelomeres possess a higher level of copy number variants (CNV) accumulation than those from the internal chromosomal cores and non-reciprocal exchanges and duplications among subtelomeric regions appear to be widespread among eukaryotes (Eichler and Sankoff, 2003), which supported our findings. We subsequently scanned the homologous ARSs of these 20 ARSs among 104 *S. cerevisiae* strains (Supplementary Table 2), and we found that the most conserved pairs among 104 strains are two pairs internal ARSs compared with those of subtelomeric ARSs. One is the pair of ARS 810 and ARS 811 closed to the tandem array of CUP1 that are associated with resistance to the toxicity of copper (Fogel and Welch, 1982). The other is the pair of ARS1200-1 and ARS 1200-2 known as rARSs (Miller and Kowalski, 1993) that are associated with yeast life span (Kwan et al., 2013). These results suggested that compared to similar ARS pairs distributed in inter-chromosomes, similar ARS pairs located in intra-chromosomes prefer to be shared between strains.

#### Population Genomic Analysis of ARSs Among 104 Yeast Genomes

The 104 *S. cerevisiae* strains that we focused on in this study showed broad genotypic and phenotypic diversity (Strope et al., 2015; Peter et al., 2018). What is the extent of distribution of replication origins in these *S. cerevisiae* strains with various phylogenetic distance? Therefore, we built a non-redundant dataset with a total of 520 ARSs based on ARSs annotation of *S. cerevisiae* S288C from SGD and supplemented with the confirmed ARSs from OriDB and DeOri database (Supplementary Table 4).

A marked similarity between two nucleotide sequences may reflect the fact that they come from the same ancestral sequence driven by evolution (Petsko and Ringe, 2004). By mapping ARS sequences from the non-redundant dataset to 104 *S. cerevisiae* genomes, we determined the homologous ARSs in each chromosome among these yeast strains. Even though the strict alignment conditions had been set, plentiful homologous ARS sequences were found among various yeast genomes and the majority of the homologous ARSs were mapped to the chromosome of the corresponding ARS of the non-redundant dataset. We measured the proportion of homologous ARSs in all strains within the species of *S. cerevisiae* and displayed it in heat map plot (Supplementary Figure 3), illustrating that the conservation profile of ARSs is not evenly distributed along the chromosomes. It should be noted that only the homologous ARSs located in the corresponding chromosome of the 520 ARS dataset were collected. As for the non-unique ARSs, their chromosomal regions were also taken into consideration. According to the number of homologous ARSs in 104 budding yeast strains, we defined those ARSs existing in more than 90% of the yeast strains as conserved ARSs, and the rest as non-conserved ARSs. We found 430 conserved ARSs accounting for 82.7% of the ARSs from the dataset, and these ARSs are not only conserved in sequence but also relatively conserved in the chromosomal position among various *S. cerevisiae* genomes (Supplementary Figure 4A), which likely served as the organizational framework for *S. cerevisiae* genomes. Although large-scale structural variants might exist in the chromosome XII of different yeast strains, the relative position between ARSs in the fragments with structural variants are conserved. Interestingly, about 80% of ARSs located in the subtelomeric regions are non-conserved ARSs. The number of homologous ARSs from subtelomeres is less than those from internal chromosomal regions (one-side Mann–Whitney *U* test, *p*-value < 0.01). Subtelomeric regions profoundly contribute to genetic and phenotypic diversity, which recognized as peculiarly dynamic regions of chromosomal evolution (Eichler and Sankoff, 2003; Dujon, 2010; Yue et al., 2017). These areas with rampant genomic rearrangement are the hotspots of reciprocal translocations (Eichler and Sankoff, 2003), which could interpret that ARSs located in subtelomeric regions possess lower conservation than those located in internal chromosomal regions. Subtelomeric regions showed a strong relevance of rapid adaptation to novel niches (Brown et al., 2010; Bergström et al., 2014; Yue et al., 2017), and were known as the hot spots of genetic variation (Peter et al., 2018), which helps accelerate genome evolution and divergence (Cohn et al., 2006). The distribution of non-conserved ARSs in subtelomeres of different *S. cerevisiae* strains may reflect the strain specificity during genome evolution.

We also performed the pan-genome analysis of ARSs (pan-ARSs) among 104 *S. cerevisiae* strains by PanGP (Zhao et al., 2014) software. The pan-ARSs size curve illustrated closed pan-ARSs (Supplementary Figure 5). Since we adopted limited ARSs pool to map to yeast strains, it seems that the additional strains could not provide new ARSs to *S. cerevisiae* pan-ARSs. The result showed that a small number of yeast strains are sufficient to cover the majority of *S. cerevisiae* pan-ARSs. Core ARSs represent the ARSs exist in all strains of *S. cerevisiae*. The core ARSs curve showed that the size of core ARS approached to a constant value, suggesting that these 183 core ARSs might serve as the organizational framework for the *S. cerevisiae* genome.

The total number of homologous ARSs corresponding to the chromosome of ARS from non-redundant dataset for each strain was calculated, and the amount of data we obtained was sufficient for subsequent analysis. Based on the geographic and environmental origins of yeast strains (Supplementary Table 5), we classified the strains into several subsets. By illustrating the distribution of homologous ARSs in each subset (Supplementary Figure 6), we found that the average number of homologous ARSs in each category are relatively similar, however, due to distinct ecological niche and various degree of human association of the isolated strains, the data fluctuation range are various from each class, which may underline a key role of human-driven activities in shaping the distribution of ARSs in *S. cerevisiae* (Strope et al., 2015; Peter et al., 2018).

In the process of DNA replication, the ORC is recruited to replication origins, followed by the binding of CDC6 (cell division cycle 6) and CDT1 (Cdc10-dependent transcript 1) as well as loading of the MCM helicase complex, which formed the pre-RC (pre-replication complex) proteins (Fragkos et al., 2015). The pre-RCs would bind to all potential origins, however, potential replication origins are in excess and only a small fraction of assembled pre-RCs will be activated at each cell cycle. In addtion, the activation of pre-RCs does not occur simultaneously. Some are fired in the early S phase, and others are activated in the mid or late S phase (Méchali, 2010). The number of corresponding homologous ARSs found in different yeast strains showed the conservation of ARS within the species of *S. cerevisiae*. To analyze the correlation coefficient between the ARS conservation and the replication fire time, the data of the replication time (Raghuraman et al., 2001) together with the number of homologous ARSs among *S. cerevisiae* strains were adopted. The result showed that the conservation of ARSs was non-randomly associated with replication time, the Pearson correlation between the conservation of ARSs and replication time shows the value of −0.484 (*p*-value < 0.01). By comparing the different replication time between the conserved ARSs and non-conserved ARSs (Supplementary Figure 7), we found that the higher conservation of ARSs, the earlier it might initiate (the pairwise Wilcox.test, *p*-value < 0.001). Combining with the previous findings, we could conclude that in the species of budding yeast, the ARSs biased toward the subtelomeric regions tends to possess weaker conservation and later replicated fire time, which was consistent with the previous conclusion that subtelomeric regions generally possess late DNA replication and low levels of transcription (Barton et al., 2003; Yamazaki et al., 2013). We could also infer that those conservative and earlier replicated replication origins may possess more vital missions than others in chromosomes, for instance their neighboring genes have the priority to early replicate to maintain the growth of yeast strains.

### Functional Analysis of Genes Adjacent to Replication Origins in *S. cerevisiae* Genomes

#### Genes Adjacent to ARSs in *S. cerevisiae* S288C Reference Genome

In bacteria, the distribution of *oriC*s and its corresponding adjacent replication-related genes such as *dnaA*, *dnaN* or *gidA* are highly conserved among different phyla and around 43% of the *oriC*s are biased close to *dnaA* among a total of 2740 bacterial chromosomes distributed in various phyla (Luo et al., 2018). The relationship between the *oriC* and adjacent replication-related genes has been successfully applied to predict the location of *oriCs* in bacterial chromosomes (Gao and Zhang, 2008). In archaea, replication origins are found to locate next to *cdc6/cdc1* (Norais et al., 2007). It is worth surveying the distribution profile of eukaryotic replication origins and their corresponding adjacent genes.

It is generally accepted that the locations of replication origins are exclusively restricted to intergenic regions in eukaryotes (Brewer, 1994; Gilbert, 2001). Here we extracted protein-coding genes adjacent to replication origins of the well-annotated reference genome of *S. cerevisiae* S288C. In recent years, studies on minimal ARS (miniARS) in yeast have been reported (Liachko et al., 2013; Tsai et al., 2014). However, research on systematically and accurately identifying the precise boundaries of minimal functional replication regions have not been performed due to a large number of replication origins in yeast chromosomes. Please note that the boundaries of the ARSs used in this study are all collected from the original literature, which may be not confined to the minimum essential regions.

According to *S. cerevisiae* reference genome annotation, we classified the replication origins based on the positional relationships between ARSs and their adjacent genes in the chromosomes. It is defined as the intergenic ORI if there is no intersection between replication origin and its corresponding adjacent protein-coding genes, otherwise as the intersected ORI that the replication origin sequence partially or completely overlaps the adjacent protein-coding genes (Supplementary Figure 8). The result showed that the intergenic ORIs account for 68.18% of known replication origins of *S. cerevisiae* S288C. Distance distribution among ARSs and their adjacent protein-coding genes showed that their interval distances are mainly less than 1000 bp (Supplementary Figure 9). Although there are 112 intersected ORIs with the average length of 395 bp, about 55.35% of their overlapped segments are less than 30% of their own lengths. Since the ACS element is an essential and conservative element in ARSs, we subsequently scanned the overlapped segments between the intersected ORI and its overlapping protein-coding genes using the matrix profile of ACS motif (Supplementary File 1). The result showed that there are 40 overlapped segments contain ACS motif, suggesting the overlapped segments may be important for these ORIs. We also identified the repeats in ARSs executed by REPuter (Kurtz et al., 2001) program (options:./repfind -c -f -p -r -l 8 -best 50 -h 0 –s) with the *e*-value cutoff of 5e-2. We found that the majority (92.90%) of ARSs contain repeats (Supplementary Table 3). For intergenic ORIs, repeats (average AT content of 91.10%) are generally characterized by continuous A base, continuous T base, or alternating repeats of A and T base (Supplementary Figure 10). However, for the overlapped segment with ACS motif between the intersected ORI and its overlapping protein-coding gene, repeats in these segments (average AT content of 83.52%) are biased to possess higher GC content (Supplementary Table 3), which suggest that the sequence composition of the overlapped segment between the intersected ORI and its overlapping gene may be constrained by the gene composition.

For *S. cerevisiae*, the effectiveness of ARSs is various, and restraining the initiation of certain ARSs could affect the expression of neighboring genes. Histone gene pairs (HTA1-HTB1, HHT1-HHF1) are closely positioned to replication origins (ARS428, ARS209) in *S. cerevisiae* S288C genome. Inactivation of ARSs that are proximal to HTA1–HTB1 gene pairs significantly delayed replication of HTA1 and HTB1, resulting in halving the expression of histone genes (Muller and Nieduszynski, 2017). The delay in replication of centromeric regions (including ARS919 and ARS920) contributes to chromosome instability (Natsume et al., 2013). Nevertheless, *S. cerevisiae* with multiple origin deletions (ARS600, ARS601/2, ARS603, ARS603.5, ARS604, ARS506, and ARS606) in chromosome VI can replicate relatively normally without detectable growth defects (Dershowitz et al., 2007). Essential genes are those indispensable for the survival of an organism (Giaever et al., 2002), and we found 47 intersected ORIs overlapped with essential genes (data from DEG database^7^) (Gao et al., 2015; Supplementary Table 1). Any genetic variation occurred in the overlapped region may cause changes in both replication origin and the essential gene, which may disturb the stability and integrity of genomes and even threaten the viability of yeast cells.

Subsequently, in order to assess which factors could determine the difference between the intergenic ORIs and the intersected ORIs, principal component analysis (Supplementary Figure 11) was conducted by integrating the comprehensive features of ARSs in *S. cerevisiae* S288C including length, GC content, the positional relationship with the adjacent gene, relative chromosomal position, the number of homologous ARSs we obtained among *S. cerevisiae* species as well as replication time [data from Raghuraman et al. (2001)] and gene expression profiles of *S. cerevisiae* [data from Arava et al. (2003)]. The intergenic ORIs and intersected ORIs showed relatively visible distinction in the PCA score plot. The average expression of genes adjacent to intersected ORI was significantly lower than that of genes adjacent to intergenic ORI (one-side Mann–Whitney *U* test, *p*-value < 0.05). We guessed that replication-related proteins that bind or be recruited at replication origins may interfere with the expression of overlapping genes, and the influence of expression of overlapping genes has to be considered in space as well as time, because only a subset of origins are activated during every cell cycle and the activation of replication origins may vary according to the cell fate or environmental conditions (Méchali, 2010; Fragkos et al., 2015). However, the comparison was only based on the inference of statistical results, and the specific relationships between replication origins and their adjacent genes require more detailed experimental studies. We found that most of the above mentioned factors were linearly uncorrelated to the ORIs that possessed the various positional relationship with the adjacent gene, but the intergenic ORIs and the intersected ORIs could be roughly distinguished through the PCA analysis if these factors were comprehensively considered.

#### Genes Adjacent to Conserved ORIs Among 104 *S. cerevisiae* Genomes

In the study of population genomic analysis of ORIs among 104 yeast genomes, we identified 430 conserved ARSs from the ARSs dataset. Based on the homologous ARSs of various yeast strains, we extracted their adjacent genes from the corresponding yeast genomes. According to the number of genes located next to each of the corresponding conserved ORIs in 104 budding yeast strains, we defined the genes existing in more than 90% of the yeast strains as conserved adjacent genes, and the rest as non-conserved adjacent genes. As a result, a total of 662 conserved adjacent genes were collected and 27.64% of them belong to essential genes based on DEG database (Gao et al., 2015; Supplementary Table 4). We also found that the conserved adjacent genes relatively conserved in both chromosomal position and orientation among the various *S. cerevisiae* genomes (Supplementary Figure 4B), which suggested that the adjacent relationship between ARSs and their corresponding genes are conserved in chromosomes among 104 yeast strains.

In bacteria, the replication-related genes such as *dnaA* and *dnaN*, are highly conservatively close to *oriC*s (Luo et al., 2018). Likewise, genes involved in the initiation of DNA replication in *S. cerevisiae* are found to conservatively locate next to the replication origins, such as *orc3*, *mcm2*, *mcm4*, *mcm6*, and *cdc45* (Supplementary Table 4). During G1 phase, ORC complex recognizes and bind sequence-specifically to ACS in the presence of ATP (Bell and Stillman, 1992; Watson et al., 2013). And helicase-loading proteins, CDC6 and CDT1, are recruited to load MCM2–7 complexes onto the replication origin (Bell and Labib, 2016). During S phase, loaded helicases are activated by CDK (cyclin dependent kinase) and DDK (Dbf4-dependent kinase). Those two factors, CDC45 (cell division cycle 45) and GINS (Go, Ichi, Ni, and San) complex are tightly associated with MCM2-7 at replication forks to form the activated helicase called the CMG complex (CDC45–MCM–GINS) (Moyer et al., 2006; Pacek et al., 2006). Then the CMG complex would be well assembled and activated to unwind the double-stranded DNA and start to initiate DNA synthesis (Fragkos et al., 2015).

**FIGURE 2:**
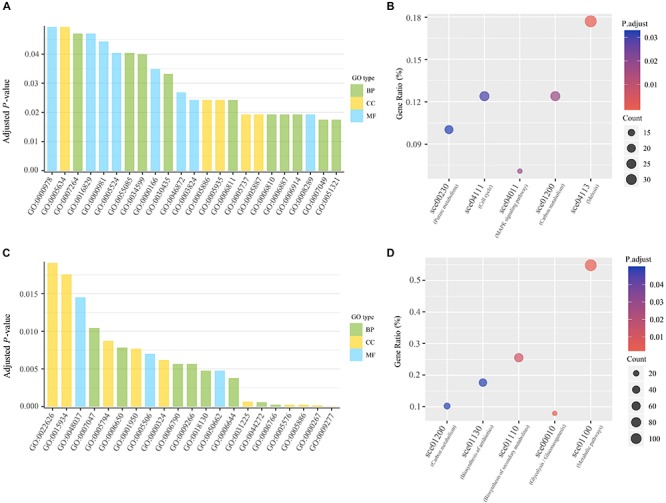
Functional analysis of genes adjacent to replication origins. **(A)** GO enrichment analysis of 662 conserved genes adjacent to conserved ARSs. GO terms with the adjusted *p*-value ≤ 0.05 are shown. The statistical significance was assessed by Fisher’s exact test with False discovery rate (fdr) correction. BP for the biological process; MF for molecular function; CC for the cellular component. **(B)** Scatterplot for significantly enriched KEGG pathways of 662 conserved genes adjacent to conserved ARSs. KEGG pathways with the adjusted *p*-value ≤ 0.05 are shown. The statistical significance was assessed by Fisher’s exact test with fdr correction. The size and color of dots represent the gene number and the adjusted *p*-value, respectively. Gene ratio is the proportion of enriched genes among all conserved genes neighboring ORIs. **(C)** GO enrichment analysis of genes adjacent to non-conserved ARSs. **(D)** Scatterplot for significantly enriched KEGG pathways of genes adjacent to non-conserved ARSs.

We found that the conserved adjacent genes were significantly enriched in twenty-three GO terms and five KEGG pathways related to DNA binding, enzyme activity, transportation and energy, including sequence-specific DNA binding, nucleotide binding, lyase activity, catalytic activity, ion transport, transmembrane transport and ATP binding (Figures 2A,B and Supplementary Table 6). Obviously, these GO terms and KEGG pathways are strongly correlated with the process of DNA replication. Due to the advantage in chromosomal position, the genes adjacent to replication origins are preferentially replicated after the double-stranded DNA start unwinding. We could infer that replication origin neighboring genes involved in DNA binding, enzyme activity, transportation, and energy might have a higher priority to replicate. It is likely that the aggregation of ORI and its conserved adjacent genes enable the gene-encoded products to be more effectively involved in the DNA replication initiation in the localized cellular space. It is possible that these preferential replicated genes enable normal and efficient DNA replication and possibly make it orderly organized in protein-DNA and protein-protein interactions, which may guarantee stable operations in the yeast cell cycle. With regard to non-conserved ARSs, also considered as strain-specific ARSs, we found that these genes are significantly enriched in response to environmental stress (such as temperature and drug), metabolites biosynthetic process and biosynthesis of antibiotics (Figures 2C,D and Supplementary Table 7). We speculated that the preferential replication related to these adjacent genes is likely to provide raw materials for the active metabolism of yeast strains, and may enhance the adaptability of the strains to survive in the environmental stress.

## Conclusion

In this study, we comprehensively analyzed features of replication origin sequences of *S. cerevisiae* from genome-wide and population genomics perspectives. We conducted the data-analytic work for investigating the similarities and genomic positions of the ARS sequences among the diverse budding yeast strains obtained from various ecological and geographical backgrounds. We also performed a characterization of the genes that are adjacent to the conserved and non-conserved ARSs among the 104 yeast strains. These results presented here may provide insights into the replication mechanism of *S. cerevisiae* and facilitate the development of algorithms for further prediction of replication origins in budding yeast genomes. For examples, the conserved ARS-adjacent genes should be taken into consideration in the design of prediction algorithms, just like Ori-Finder considering the conserved *oriC*-adjacent genes (such as *dnaA*, *dnaN*, and *gidA*), which would make the prediction more robust and reliable. In addition, as modular parts, the core ARSs and their conserved adjacent genes might provide a useful reference for the rational design of replication origins for the synthetic *S. cerevisiae* genome. However, the conserved ARSs and their corresponding adjacent genes are obtained based on sequence alignment and statistical results, whereas the biological significance of the positional conservation of the replication origins and their adjacent genes requires more detailed experimental proof. Since DNA replication is one of the highly conserved processes of eukaryotic cell, and almost all the proteins related to the DNA replication in yeast correspond to a single ortholog in humans and other eukaryotic species (Bell and Labib, 2016), the features and rules of DNA replication initiation found in *S. cerevisiae* genomes may be extended to higher eukaryotes.

## Author Contributions

DW conducted the data analysis and drafted the manuscript. FG supervised the study and revised the manuscript. Both authors read and approved the final manuscript.

## Conflict of Interest Statement

The authors declare that the research was conducted in the absence of any commercial or financial relationships that could be construed as a potential conflict of interest.

## Footnotes

1 ftp://ftp.ncbi.nih.gov/genomes/all/

2 ftp://ftp.yeastgenome.org/sequence/S288C_reference

3 http://yeastmine.yeastgenome.org

4 http://cerevisiae.oridb.org/

5 http://tubic.org/deori/

6 ftp://ftp.uniprot.org/pub/databases/uniprot/current_release/knowledgebase/complete

7 http://tubic.org/deg/
