# Supplementary material for: Comprehensive Analysis of Replication Origins in Saccharomyces cerevisiae Genomes
Source: Front Microbiol. 2019 Sep 13;10:2122. doi: 10.3389/fmicb.2019.02122 (PMC6753640; doi:10.3389/fmicb.2019.02122)
Supplement: Supplementary file 1 [file Data_Sheet_1.docx]

Supplementary Material

Comprehensive Analysis of Replication Origins in *Saccharomyces cerevisiae* Genomes

Dan Wang and Feng Gao*

*** Correspondence:** Feng Gao: fgao@edu.tju.cn

# Supplementary Figures and Tables

## Supplementary Figures

##
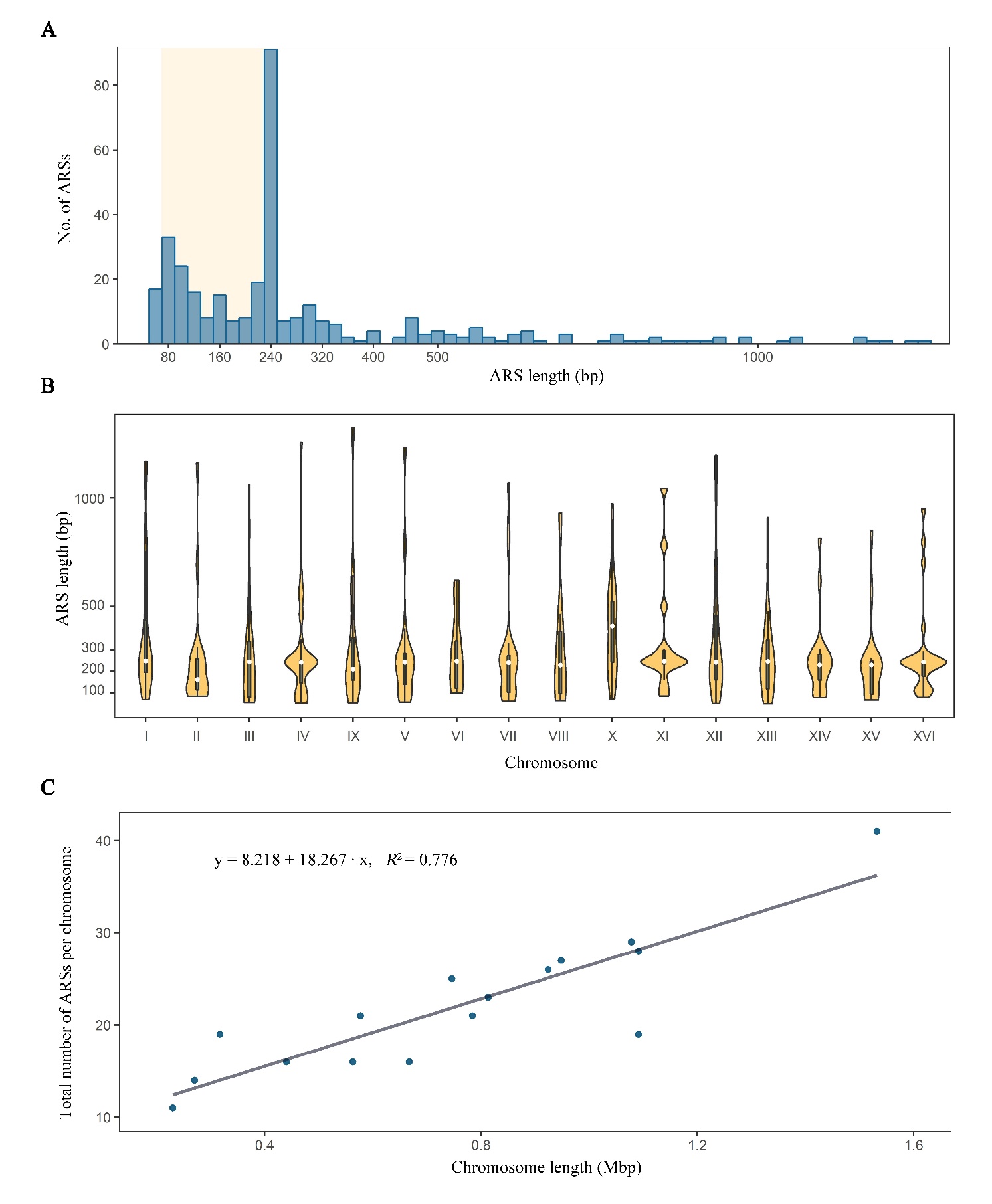


**Supplementary Figure 1**. Statistical analysis of the length and number distribution of published ARSs in reference *S. cerevisiae* S288C based on SGD database. (A) Histogram illustrates the length distribution of published ARSs of *S. cerevisiae* S288C genome. The high frequency of length is annotated by bright orange. (B) Violin plot shows the length distribution of ARSs of *S. cerevisiae* S288C in every chromosome by mirrored histograms. Center white dot, median; boxes, interquartile range (IQR); whisker, 1.5×IQR; Data points beyond the whiskers are outliers. (C) Linear regression analysis of total ARSs counts per chromosome against each length of the chromosome. The number of ARSs in every chromosome is positively correlated with chromosomal size. *R*^2^ corresponds to the square of the Pearson’s correlation coefficient.


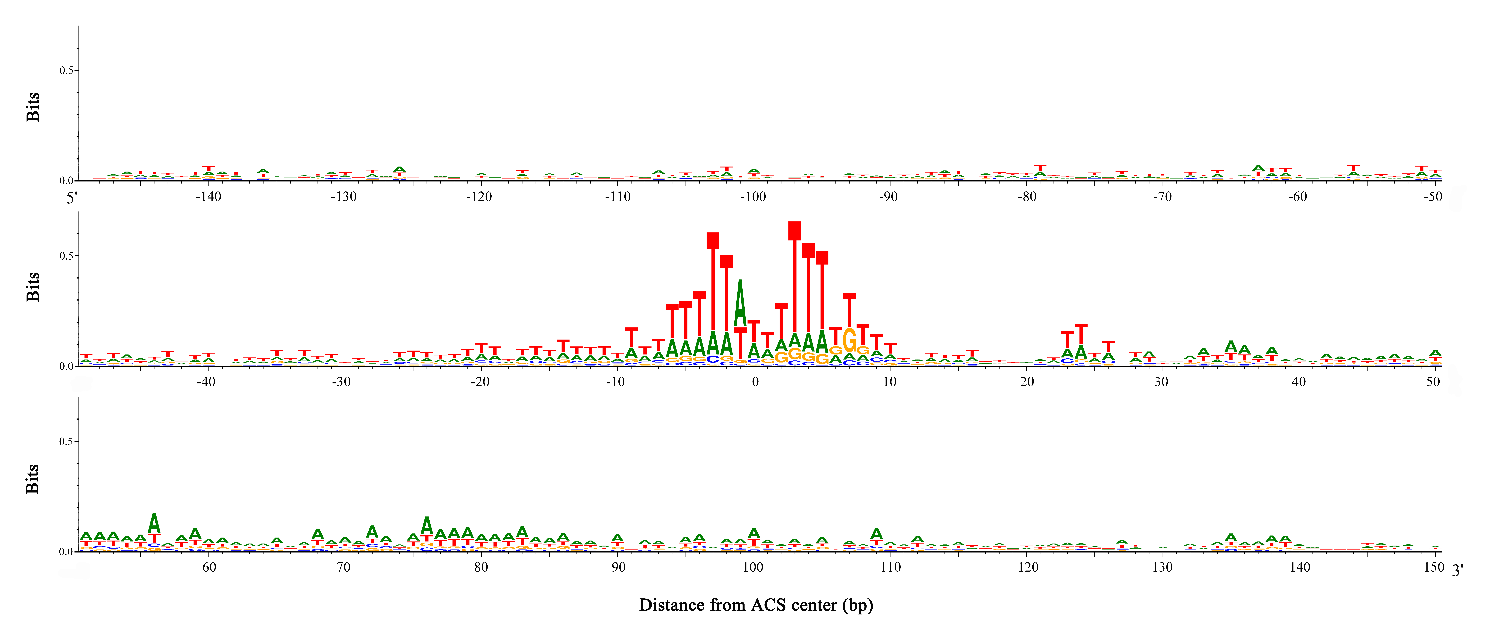
**Supplementary Figure 2.** WebLogo of ARSs containing the ACS element. The 196 published ACS sequences were downloaded from the YeastMine database populated by SGD. The WebLogo was generated using <http://weblogo.berkeley.edu/logo.cgi>. Base conservation in bits is shown for each position of the ARS sequences. The ACS element was taken as the center and appeared as the high central peak.


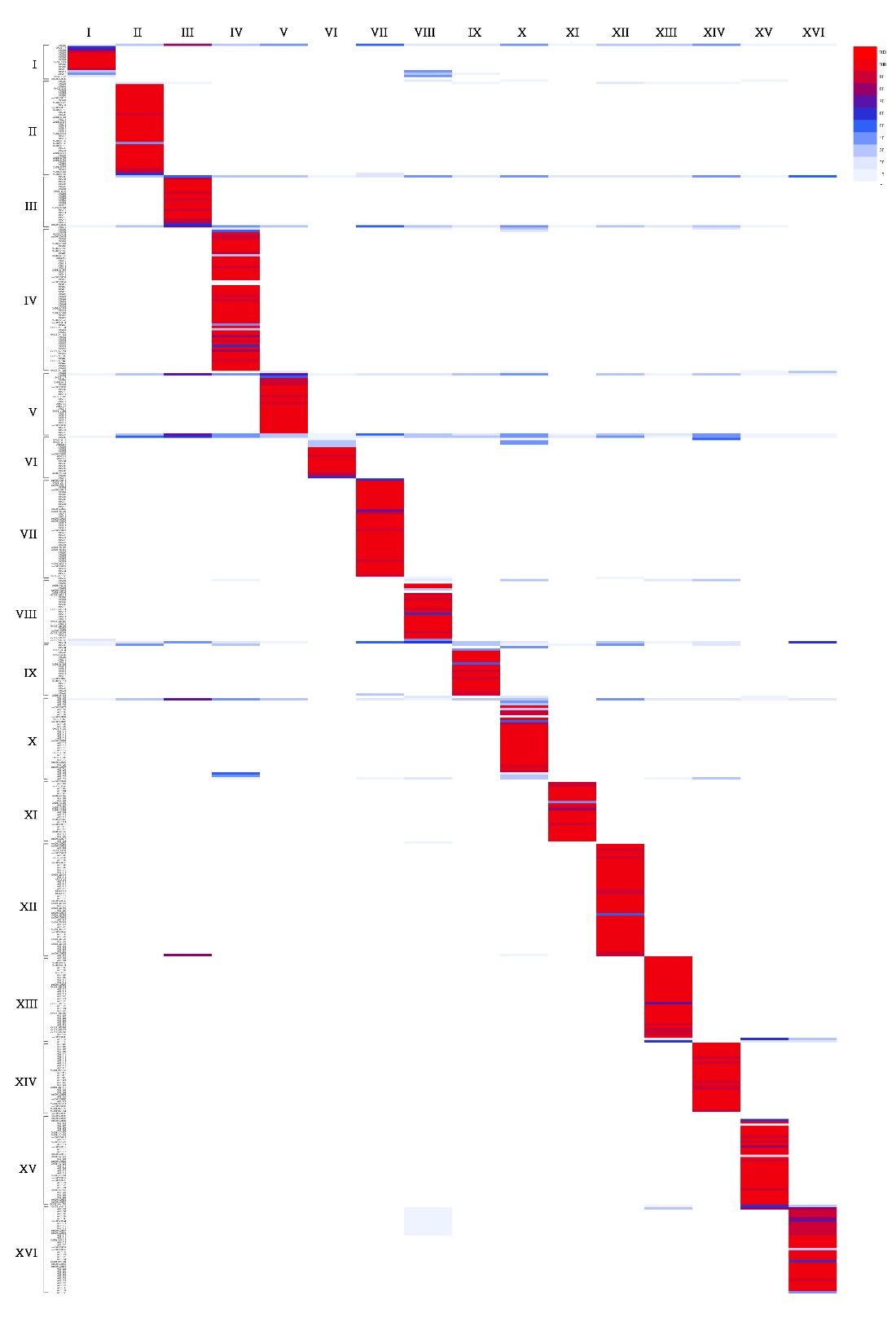


**Supplementary Figure 3.** The number of homologous ARSs of 104 yeast strains. The heat map displays the number of homologous ARSs in 104 yeast strains from high (red) to low (white). The horizontal direction shows the chromosomes in ascending order. The vertical direction indicates ARSs from the non-redundant dataset, and ARSs are arranged in ascending order according to their positions in the yeast chromosomes.

**
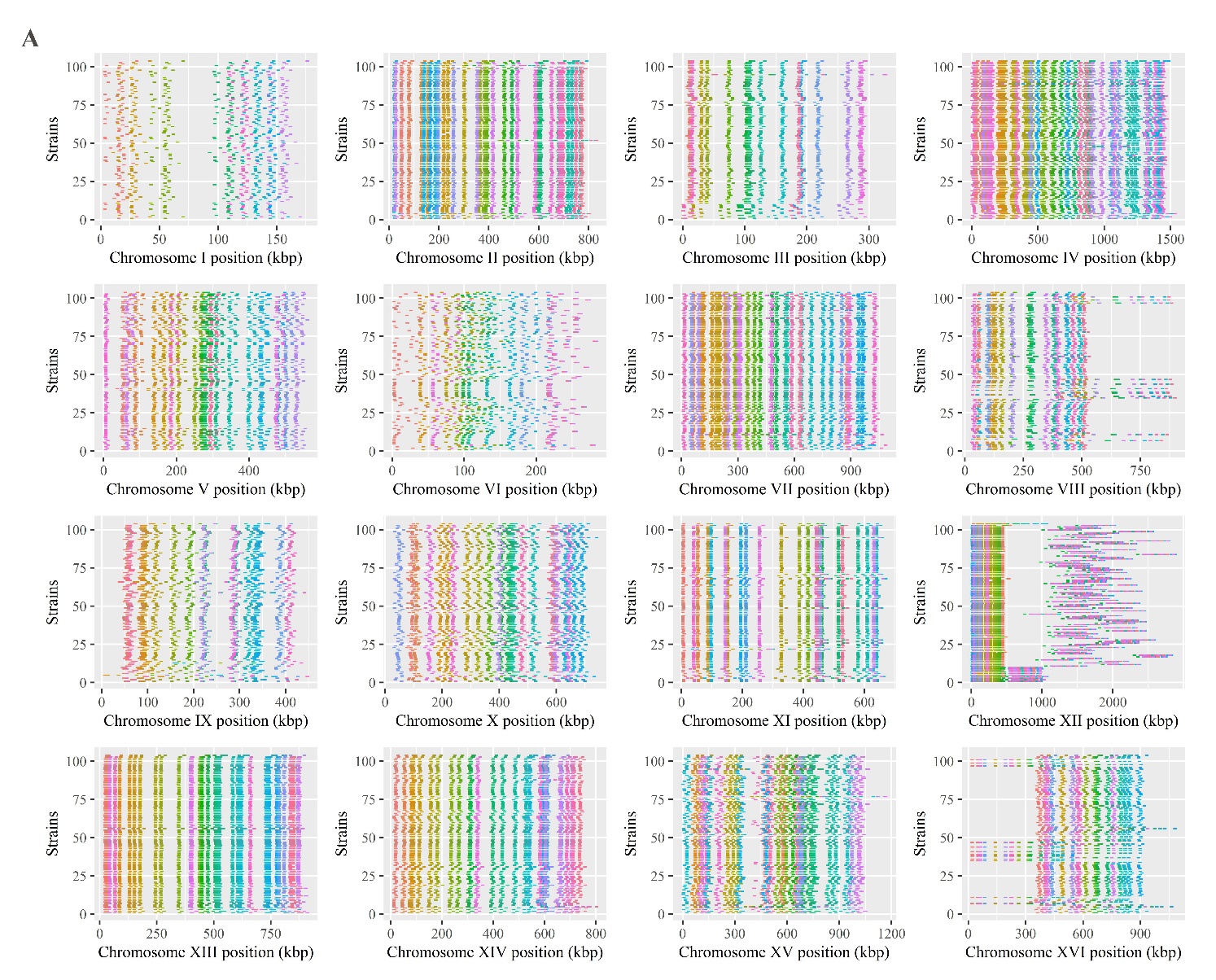
**

**
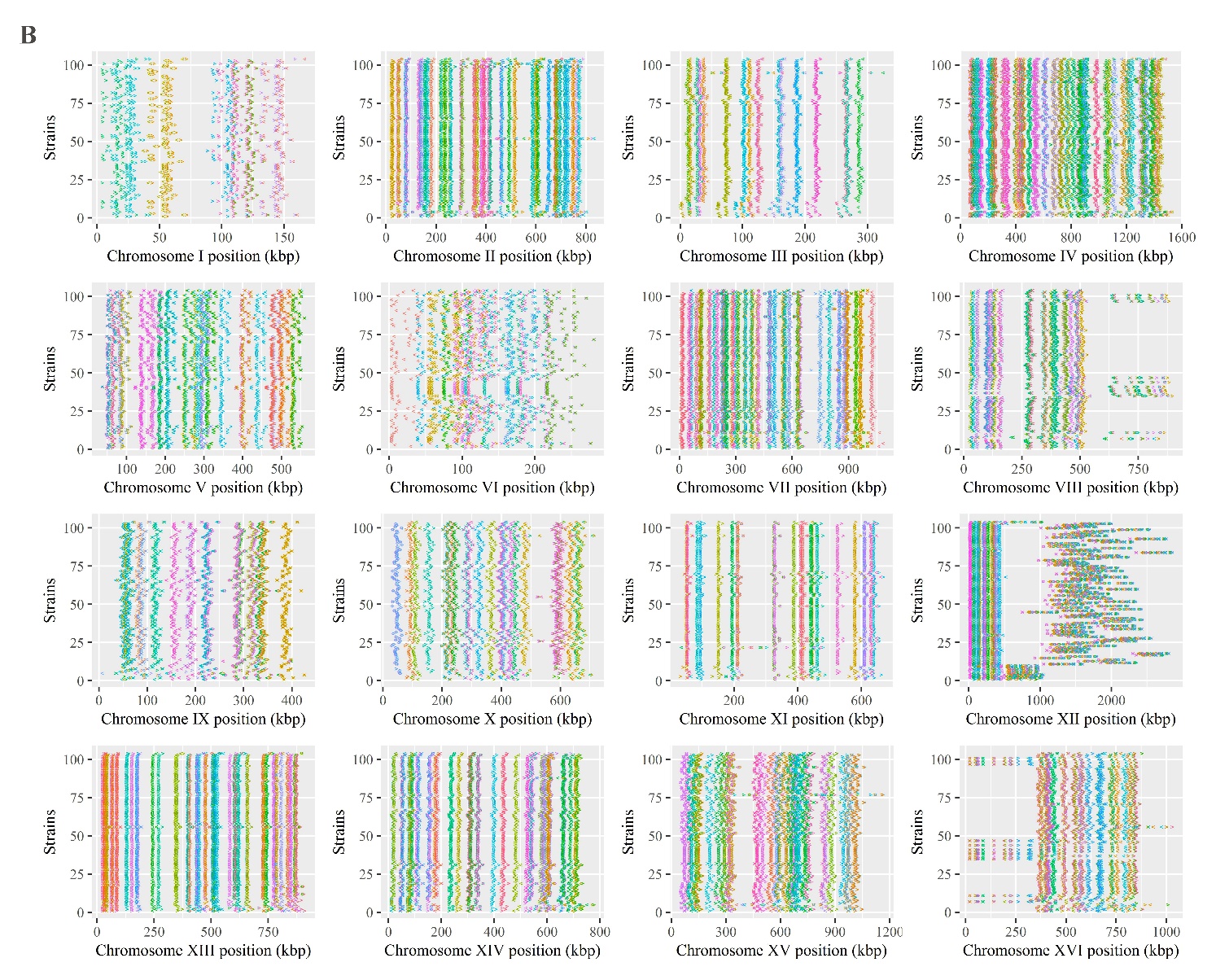
**

**Supplementary Figure 4.** (A) The distribution profile of conserved ARSs among 104 *S. cerevisiae* genomes. The x-axis represents the chromosomal position, and the y-axis indicates 104 *S. cerevisiae* strains. The blocks with different color represent various conserved ARSs of the corresponding strains. (B) The distribution and orientation of the conserved ARSs adjacent genes among 104 S. cerevisiae genomes. The x-axis represents the chromosomal position, and the y-axis indicates 104 *S. cerevisiae* strains. Arrow head indicates the orientation of conserved ARSs adjacent genes, and the arrow color represent denotes various conserved ARSs adjacent genes of the corresponding strains.

Note: Please click the hyperlink of the figures with high-resolution to see the detailed information.


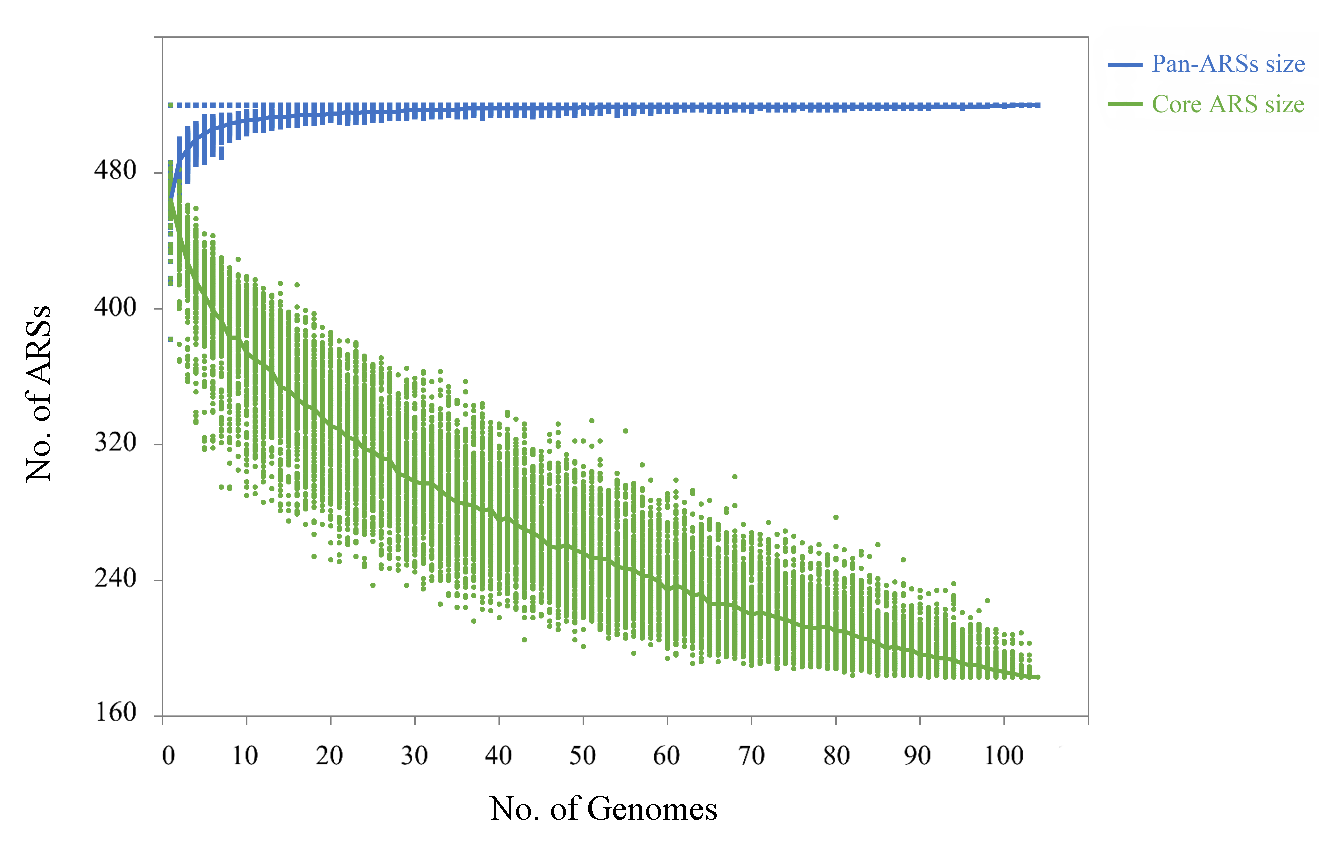


**Supplementary Figure 5.** The pan-genome analysis of ARSs (pan-ARSs) among 104 *S. cerevisiae* strains. The blue dots denote the size of the pan-ARSs for each combination of strains performed by the sampling algorithm of distance guide (DG) (Zhao et al., 2014). The blue line shows the size of *S. cerevisiae* pan-ARSs quickly saturate to a limiting value, indicating a closed pan-ARSs species. Core ARSs represents the ARSs exist in all strains of *S. cerevisiae*. The green dots convey similar information for the core ARS just as the pan-ARSs. The green line shows that the size of the core ARS approached to a constant value.


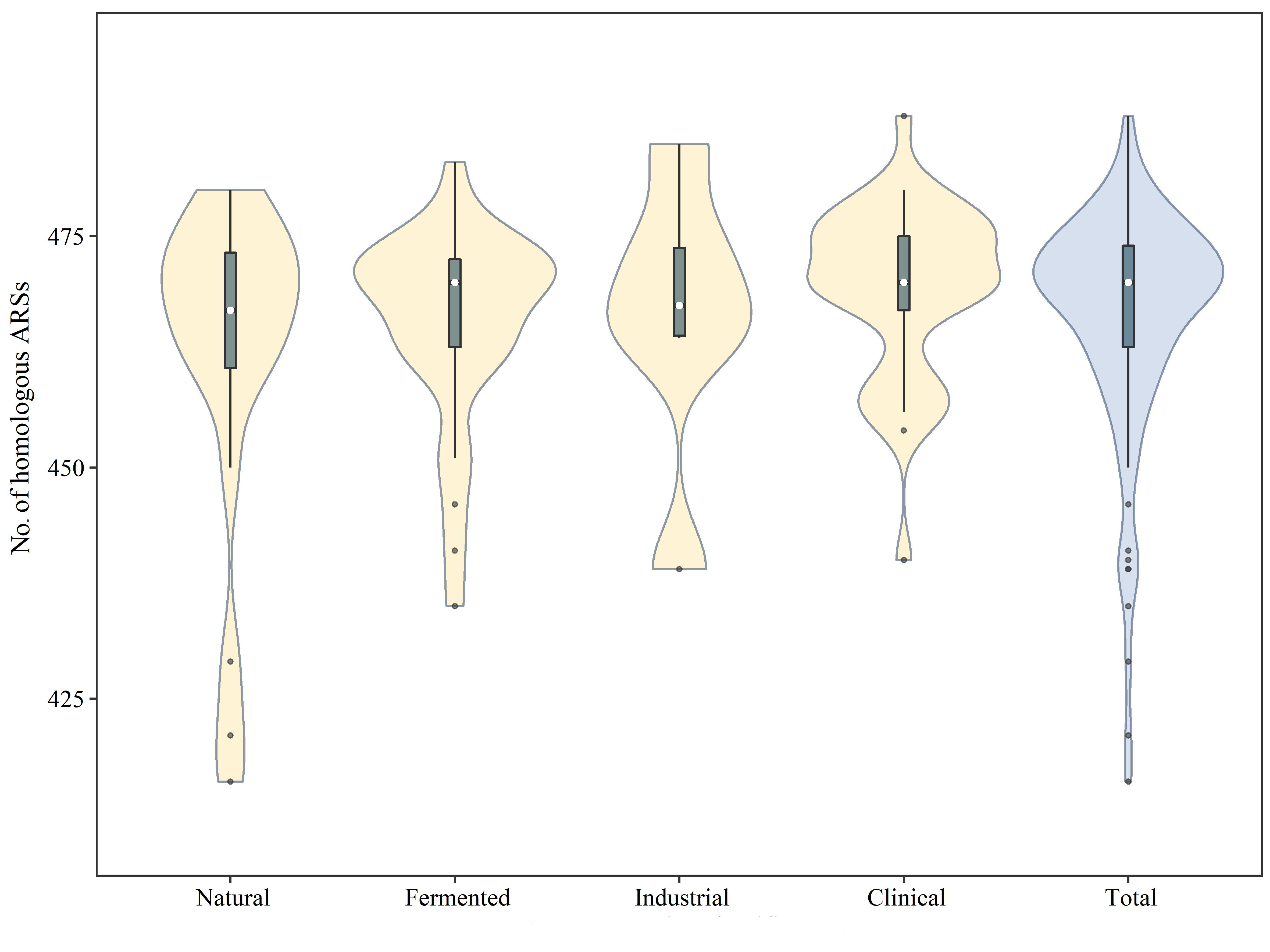


**Supplementary Figure 6.** The number of homologous ARSs of yeast strains in different ecological classification. Violin plot shows the distribution of the total number of ARSs of yeast strains in different categories by mirrored histograms. Center white dot, median; boxes, interquartile range (IQR); whisker, 1.5×IQR; Data points beyond the whiskers are outliers.


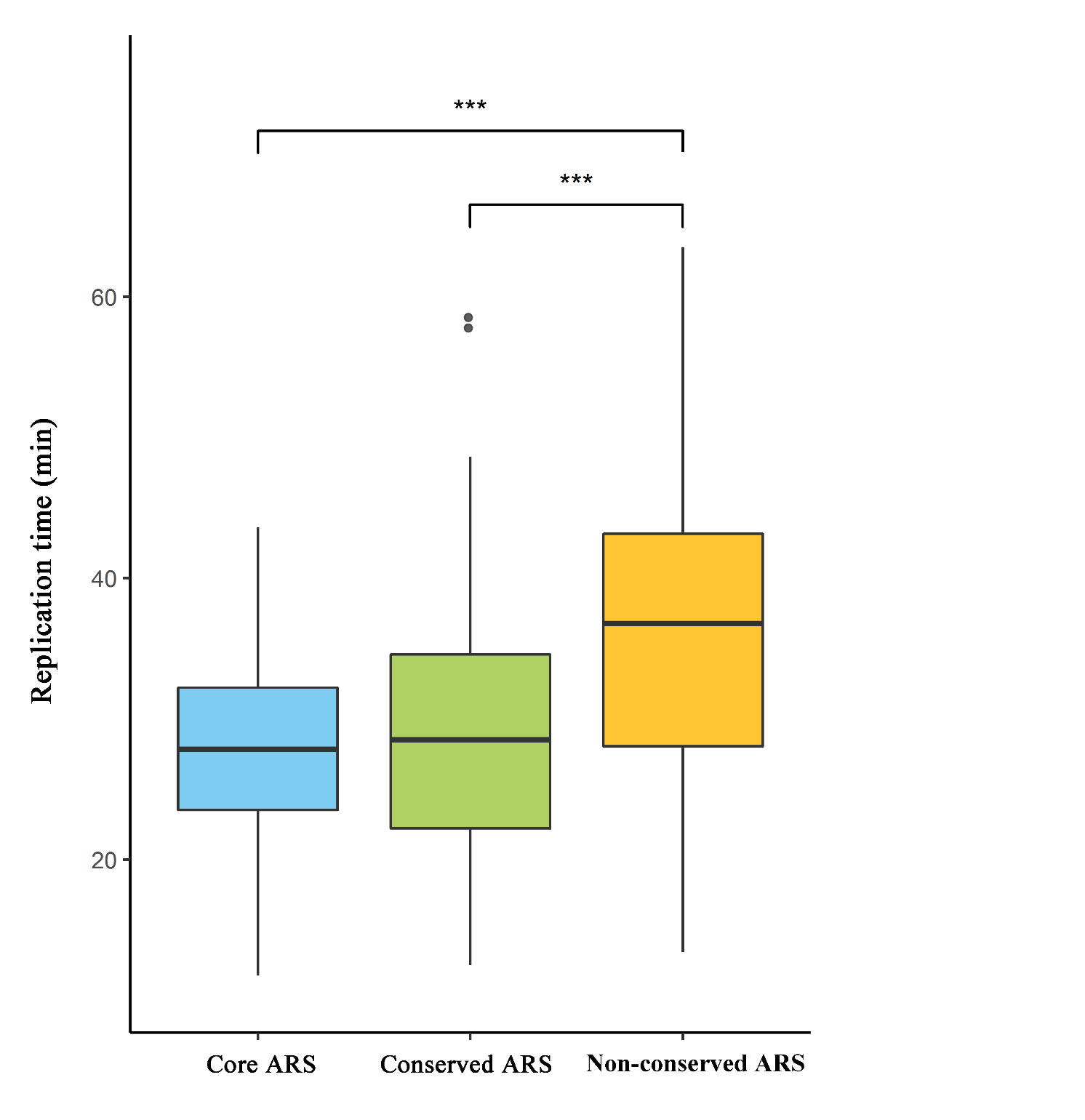


**Supplementary Figure 7.** Comparison of replication time between ARSs with various conservation in *S. cerevisiae*. Core ARSs represent the corresponding homologous ARSs exist in all strains of *S. cerevisiae*. The corresponding homologous ARSs existing in more than 90% of the yeast strains were defined as conserved ARSs, and the rest were non-conserved ARSs. Data points beyond the whiskers are outliers. Significance of the above analysis is estimated by the pairwise Wilcox.test, and “***” represents *p*-value < 0.001.


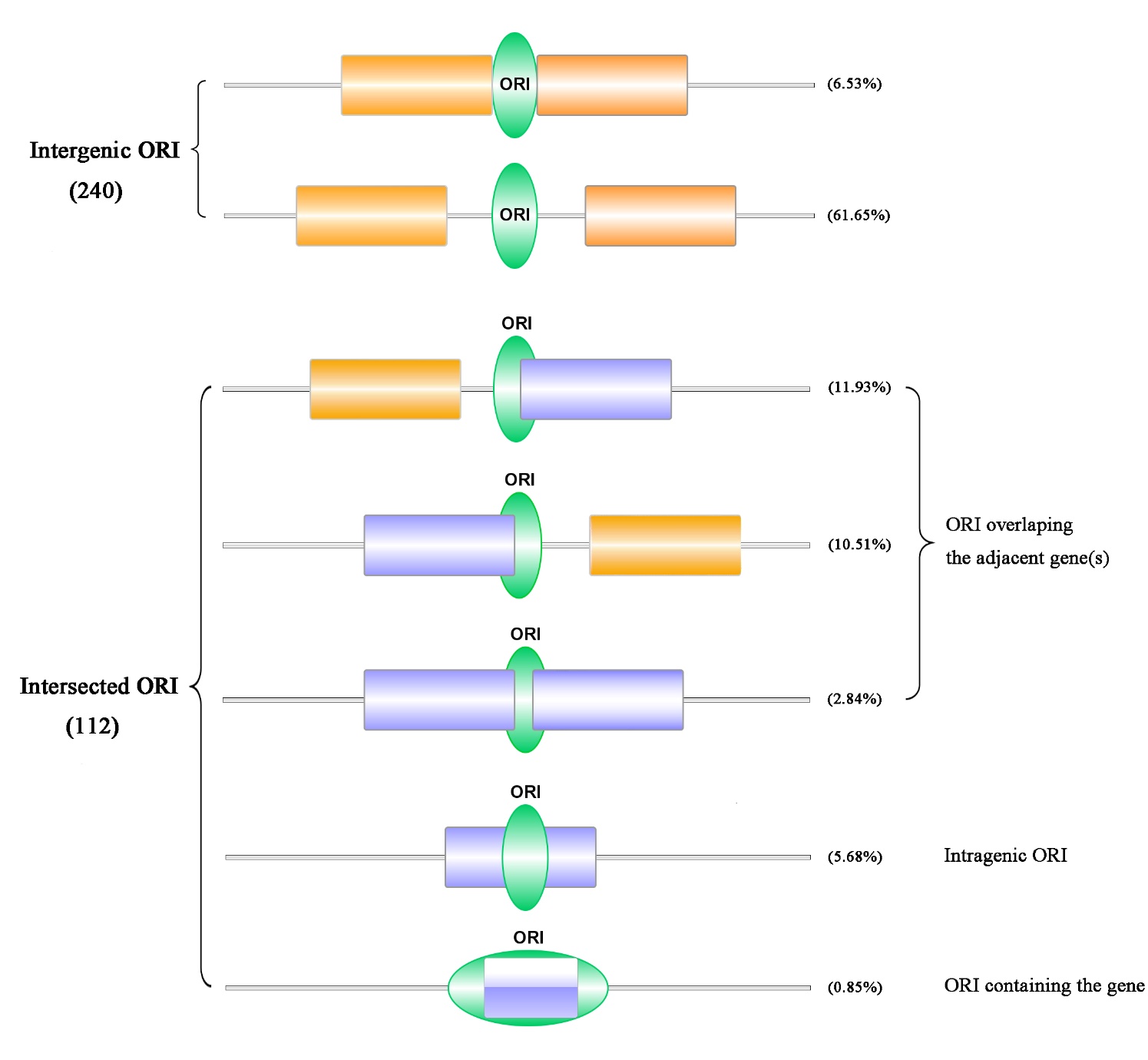
 **Supplementary Figure 8.** The distribution of ORIs and their adjacent genes in *S. cerevisiae* S288C. The green ellipse represents the replication origin, and the orange or purple rectangle stand**s** for the genes adjacent to the replication origin. The number of ARSs for each type is indicated in round brackets.

**
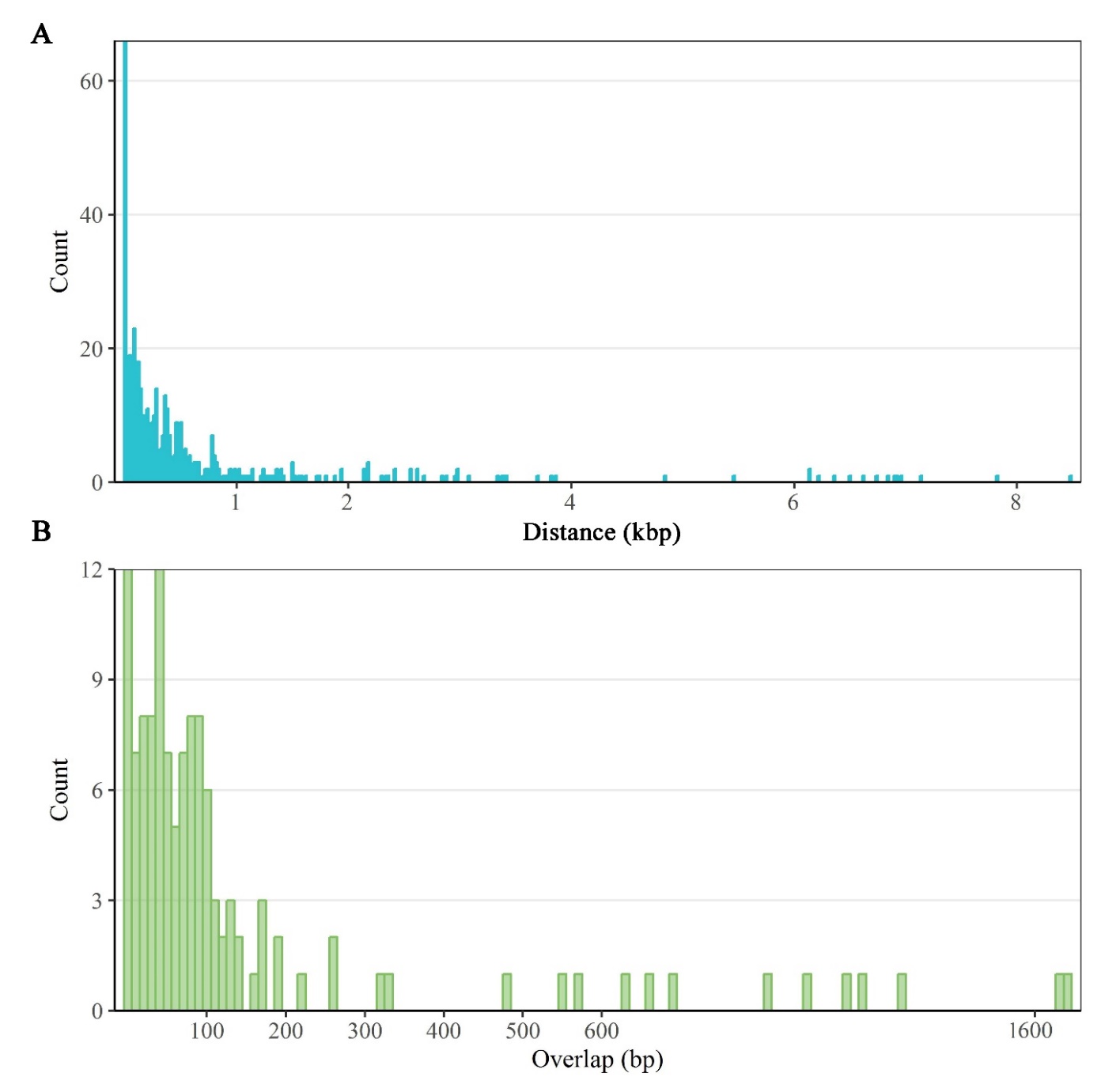
**

**Supplementary Figure 9.** Distance and overlap distribution between replication origins and their adjacent genes in *S. cerevisiae* S288C. (A) Histogram illustrates the distance distribution of intergenic ORIs and their corresponding adjacent genes. (B) Histogram illustrates the overlap distribution of intersected ORIs and their adjacent genes.


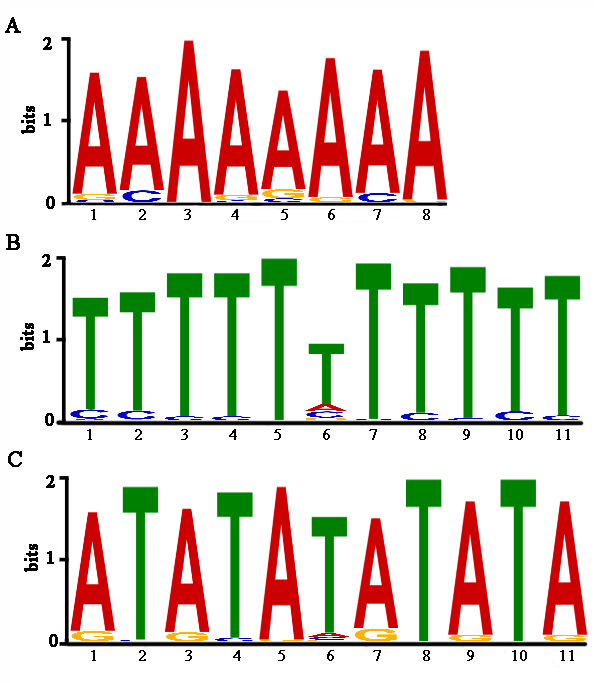


**Supplementary Figure 10**. Motifs of repeats in intergenic ORIs of *S. cerevisiae* S288C. The WebLogo plots show the base conservation of repeats in intergenic ORIs generated by the MEME Suite (Bailey et al., 2009).


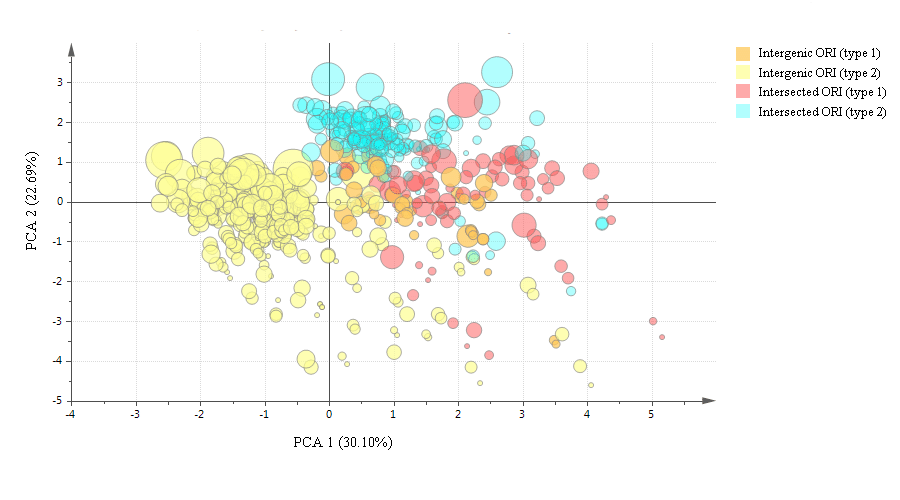


**Supplementary Figure 11.** Principal component analysis of published ARSs in *S. cerevisiae* S288C. The features of ARSs included length, GC content, the positional relationship with the adjacent gene, relative position in chromosomes, the number of homologous ARS we obtained among *S. cerevisiae* species, replication time (data from Raghuraman et al., 2001) as well as corresponding adjacent gene expression (data from Arava et al., 2003). Different color of circles represent various ARS types. The first type of intergenic ORI represents ORI directly linked to the adjacent genes, and the seconed type of intergenic ORIs are showed in yellow. The first type of intersected ORI whose overlapped segments between the intersected ORI and its overlapping protein-coding genes contains ACS motif is shown in red, and the seconed type of intersected ORIs are showed in blue. Different size of circles represent various gene expression of the nearest gene close to ARS.

## Supplementary Tables

**Supplementary Table 1**. Comprehensive information of ARS sequences in *S. cerevisiae* S288C.

Note: Please click the hyperlink of the supplementary table to see the detailed information of ARSs.

**Supplementary Table 2**. Sequence similarity analysis of ARS sequences.

Note: Please click the hyperlink of the supplementary table to see the detailed information.

**Supplementary Table 3**. Repeats in replication origins of *S. cerevisiae* S288C.

Note: Please click the hyperlink of the supplementary table to see the detailed information of repeats in replication origins.

**Supplementary Table 4**. Population genomic analysis of ARSs among 104 *S. cerevisiae* strains.

Note: Please click the hyperlink of the supplementary table to see the detailed information.

**Supplementary Table 5**. List of 104 strains with their name, geographic and environmental origin, classification, as well as the number of homologous ARS sequences among yeast strains.

Note: Please click the hyperlink of the supplementary table to see the detailed information of yeast strains.

**Supplementary Table 6**. Enrichment analysis of conserved genes adjacent to conserved ARSs.

Note: Please click the hyperlink of the supplementary table to see the detailed information.

**Supplementary Table 7**. Enrichment analysis of genes adjacent to non-conserved conserved ARSs.

Note: Please click the hyperlink of the supplementary table to see the detailed information.

**Supplementary File 1**. The matrix profile of ACS motif.

Note: We found 80% of the published 196 ACSs could be predicted by this matrix using “Bio.motifs” package included in Biopython, which can be used for subsequent research. Please click the hyperlink of the supplementary file to see the detailed information.

**Supplementary File 2A**. Similar ARS pairs distributed in *S. cerevisiae* S288C chromosomes. (A) Similar ARS pairs distributed in intra-chromosomes. (B) Similar ARS pairs distributed in inter-chromosomes.

Note: Please click the hyperlink of the supplementary files to see the detailed information.

**Reference:**

Arava, Y., Wang, Y., Storey, J.D., Liu, C.L., Brown, P.O., and Herschlag, D. (2003). Genome-wide analysis of mRNA translation profiles in Saccharomyces cerevisiae. *Proceedings of the National Academy of Sciences* 100**,** 3889-3894.

Bailey, T.L., Boden, M., Buske, F.A., Frith, M., Grant, C.E., Clementi, L., Ren, J., Li, W.W., and Noble, W.S. (2009). MEME SUITE: tools for motif discovery and searching. *Nucleic Acids Res* 37**,** W202-208.

Raghuraman, M., Winzeler, E.A., Collingwood, D., Hunt, S., Wodicka, L., Conway, A., Lockhart, D.J., Davis, R.W., Brewer, B.J., and Fangman, W.L. (2001). Replication dynamics of the yeast genome. *Science* 294**,** 115-121.

Zhao, Y., Jia, X., Yang, J., Ling, Y., Zhang, Z., Yu, J., Wu, J., and Xiao, J. (2014). PanGP: a tool for quickly analyzing bacterial pan-genome profile. *Bioinformatics* 30**,** 1297-1299.
